# Comparison of outcomes between rectal squamous cell carcinoma and adenocarcinoma

**DOI:** 10.1002/cam4.927

**Published:** 2016-10-26

**Authors:** Max S. Chiu, Vivek Verma, Nathan R. Bennion, Abhijeet R. Bhirud, Jinluan Li, Mary E. Charlton, Chandrakanth Are, Chi Lin

**Affiliations:** ^1^ Department of Radiation OncologyUniversity of Nebraska Medical CenterOmahaNebraska; ^2^ Department of Radiation OncologyFujian Cancer HospitalFuzhouChina; ^3^ University of Iowa College of Public HealthVA Center for Comprehensive Access & Delivery Research & EvaluationIowa CityIowa; ^4^ Department of EpidemiologyUniversity of Iowa College of Public HealthIowa CityIowa

**Keywords:** Adenocarcinoma, radiotherapy, rectal cancer, SEER, squamous cell carcinoma, survival

## Abstract

Large, population‐based analyses of rectal squamous cell carcinoma (SCC) have not been previously conducted. We assessed patterns of care, prognostic factors, and outcomes of rectal SCC and adenocarcinoma (AC) in population‐based cohorts. Surveillance, Epidemiology, and End Results (SEER) registry searches were performed (1998–2011), producing 42,308 nonmetastatic rectal cancer patients (999 SCC and 41,309 AC). Patient, tumor, and treatment characteristics were compared. Based on risk factors, SCC/AC groups were subdivided into low‐, intermediate‐, and high‐risk groups. Overall survival (OS) was compared between histological and risk groups using Kaplan–Meier method and log‐rank test. Multivariate logistic regression models evaluated prognostic factors for 5‐year survival. Cox regression modeling was performed on propensity‐matched data. Rectal SCC, more common in females and associated with larger tumors of higher grade, was more often treated with radiotherapy (RT) than surgery. Surgery was associated with higher OS in AC but not SCC, and RT had proportionally greater benefits in SCC. These effects of RT and surgery were retained when stratified into risk groups (particularly high/intermediate‐risk). Favorable prognostic factors for survival included younger age, non‐black race, SCC histology, size ≤3.9 cm, localized stage, lower grade, surgery, and RT. For SCC, race, tumor grade, and surgery were not prognostic factors for survival. Cox regression modeling of propensity‐matched data showed that AC histology increased risk of death versus SCC. In the largest analysis of rectal SCC to date, and in the notable absence (and unlikelihood) of prospective data, nonsurgical and RT‐based treatment is recommended.

## Introduction

As the third leading cause of cancer death among both men and women and the third most common cancer in the United States, colorectal cancer is associated with substantial morbidity and mortality in the nation's population. The rectum is the second most commonly affected site after the proximal colon, and these variations in location are also associated with differences in etiology and carcinogenesis 1. Of the malignancies affecting the rectum, over 90% are adenocarcinomas, while squamous cell carcinomas are quite rare. Nevertheless, as more of these cases are diagnosed in the coming years, evidence‐based justification of management is essential.

When compared with rectal adenocarcinoma (AC), rectal squamous cell carcinoma (SCC) occurs in approximately 0.10–0.25 per 1000 colorectal cancers 2, 3. An autopsy study of 1464 benign and malignant rectal specimens demonstrated one rectal SCC case among 423 malignant lesions 4. In 1933, Raiford reported the first case of SCC, and only a limited number of reports since then 5. Previous studies have found SCC to be a distinct entity from AC in terms of etiology, epidemiology, pathogenesis, and treatment approaches 2.

ACs are known to develop from preexisting tubular or villous adenomas via the adenomacarcinoma sequence or more rarely from dysplasia within flat mucosa, which may be associated with the development of AC in patients with hereditary nonpolyposis colorectal cancer (HNPCC) or Lynch syndrome 6. Rectal ACs tend to affect the elderly with higher incidences in men than women and with known risk factors including obesity, sedentary lifestyle, alcohol abuse, smoking, low fiber intake, family history, and inherited conditions such as HNPCC or familial adenomatous polyposis (FAP) 7. On the other hand, SCCs of the rectum are less understood and have multiple theories of pathogenesis. These include developmental theories such as malignant transformation of persistent ectopic embryonal nests of ectodermal cells, smoking‐induced de novo mutations, previous radiation exposure, chronic rectal inflammation (e.g., ulcerative colitis), infection with human immunodeficiency virus (HIV), human papillomavirus (HPV), or enteric infections such as amebiasis or schistosomiasis due to resulting squamous metaplasia 8, 9, 10, 11, 12, 13, 14, 15.

In addition to theories of pathogenesis, little is known regarding whether prognosis and treatment outcomes of rectal SCC differs from that of AC; it is unlikely that prospective studies, or even comparatively large retrospective cohorts, would be able to accumulate large volumes of patients to permit robust conclusions. Therefore, analyses of large population‐based databases such as Surveillance, Epidemiology, and End Results (SEER) are valuable for uncommon tumors such as rectal SCC. To date, such an analysis has not been reported; therefore, the objective of this study was to compare interventions, outcomes, and prognostic factors of SCC to those of AC using the SEER database.

## Materials and Methods

This study was approved by the Institutional Review Board. In order to analyze large volumes of patients with rectal SCC and AC, we utilized the SEER registry, which encompasses an estimated 28% of the United States population, including minority populations 16. A total of 47,597 patients diagnosed with locoregional rectal (site code: C20.9) squamous cell carcinoma (histology code: 8070–8077) (SCC) or adenocarcinoma (histology code: 8140–8147) (AC) between 1998 and 2011 were identified from the SEER database. Metastatic cases were not chosen, as this population usually involves treatment paradigms such as palliative chemotherapy alone (i.e., no radiotherapy/surgery). Dates earlier than 1998 displayed poor/ambiguous quality of the data/treatments, and were hence excluded. Because of the importance of the following variables in our analysis, we further excluded records with missing data on grade, surgery, and radiotherapy (RT). Thus, 42,308 patients remained. Of these, 999 patients were diagnosed with SCC and 41,309 patients with AC.

Between the two histological groups, demographic, tumor, and treatment characteristics were then collated and compared. These included age (≤65 or >65), gender, race (white or non‐white), median tumor size (<3.9 cm or ≥3.9 cm), grade (well‐moderate or poor‐undifferentiated), stage (local or regional), year of diagnosis (1998–2003 or 2004–2011), with or without surgery, and with or without RT. Cutoff values were chosen in order to balance groups' sample sizes and avoid one‐sided comparisons. Based on these candidate variables, low‐, intermediate‐, and high‐risk groups were constructed (based on number of unfavorable risk factors) for each histologic type and outcomes compared between risk groups.

The chi‐square test was used to compare the differences in proportions for the baseline patient and disease characteristics and surgery/RT receipt between the AC and SCC groups. The interval‐to‐event distributions were estimated using the Kaplan–Meier method and compared using the log rank test. A 1:1 case–control match on the propensity score with Greedy algorithms was used to balance groups by creating a cohort of patients with AC (control) that was comparable on all observed covariates (prognostic factors) to a group of patients with SCC (case) 17. Hazard ratios (HRs) and 95% confidence intervals (CIs) were calculated using Cox proportional hazards regression with variants of living status as the time metric. Stepwise elimination was used to select variables for multivariable adjusted models characterizing the association of histology (SCC vs. AC) with overall survival. A *P* < 0.05 was considered significant. All statistical calculations were performed using SAS version 9.4 (SAS Institute Inc., Cary, NC).

## Results

### Patient characteristics

As summarized in Table 1, the median age was 61 for patients with SCC and 66 for patients with AC; there were similar racial incidence. In contrast to AC, which affected females in 40% of cases, SCC affected females in 69%. Sixty‐four percent of SCC cases presented with localized stage, as compared with 47% in AC. However, 46% of SCCs were histologic grade 3/4 at diagnosis, as compared to 15% of AC cases as such.

**Table 1:** Clinical characteristics of the study population

	Adenocarcinoma*N* (%)	Squamous cell Ca*N* (%)	*P* valueChi‐square
Number of patients	41309	999	
Age			<0.0001
Median	66 (19–100)	61 (20–99)	
Age (19–65)	11051 (27)	378 (38)	
Age (66–100)	30258 (73)	621 (62)	
Gender			<0.0001
Male	24601 (60)	308 (31)	
Female	16708 (40)	691 (69)	
Race			0.113
White	34278 (83)	848 (85)	
Non‐White	7031 (17)	151 (15)	
Tumor Size Missing = 9053			0.912
Median	3.9 (0.1–10.4) cm	3.9 (0.9–8.2) cm	
>3.9 cm	17155 (53)	308 (53)	
≤3.9 cm	15516 (47)	276 (47)	
Stage			<0.0001
Localized	19898 (47)	635 (64)	
Regional	21411 (52)	364 (36)	
Grade			<0.0001
1 and 2	35170 (85)	540 (54)	
3 and 4	6139 (15)	459 (46)	
Surgery			<0.0001
Yes	36967 (89)	398 (40)	
No	4342 (11)	601 (60)	
Radiation therapy			<0.0001
Yes	24398 (59)	748 (75)	
No	16911 (41)	251 (25)	
Year of diagnosis			<0.0001
1998–2003	16246 (39)	328 (33)	
2004–2011	25063 (61)	671 (67)	

Forty percent of SCC patients underwent surgery, and 75% received RT. In AC, 89% underwent surgery, and 59% received RT. Although the majority of both SCC and AC were diagnosed between 2004 and 2011, the percent was higher for SCC (67%) than for AC (61%).

### Survival analysis

**Figure 1:**
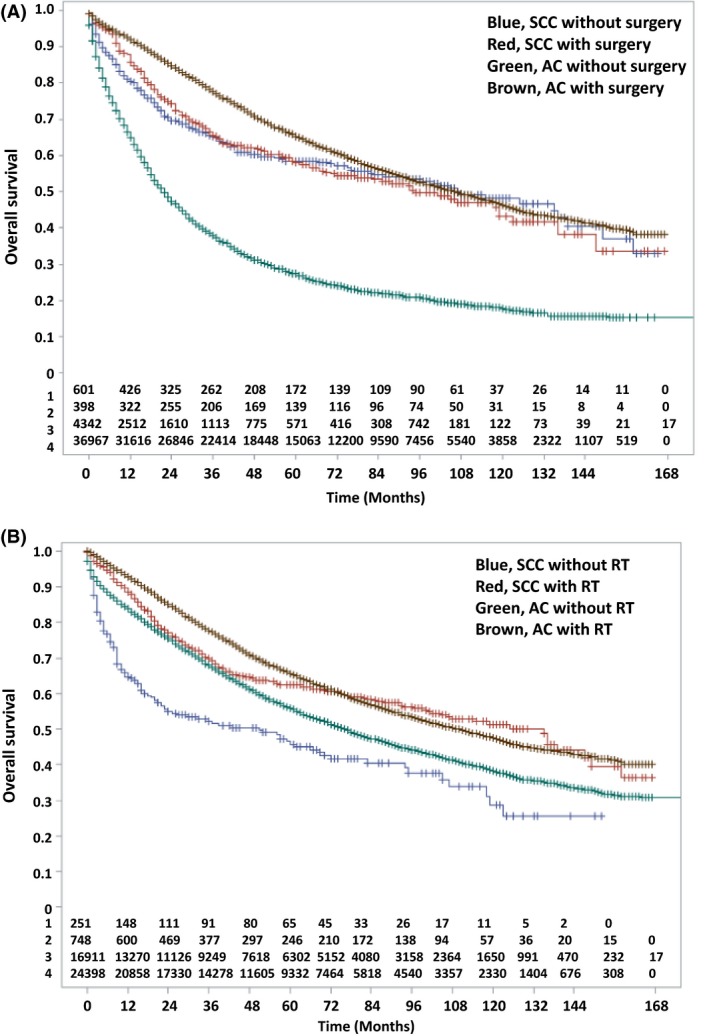
(A) Comparison of overall survival between patients with rectal SCC and AC with and without surgery. (B) Comparison of overall survival between patients with rectal SCC and AC with and without RT. SCC, squamous cell carcinoma; AC, adenocarcinoma; RT, radiotherapy.

As shown in Figure 1A, surgery improved overall survival (OS) in AC (median survival (MS) 106 months with surgery vs. 22 months without, *P* < 0.0001), but seemed to have no effect on SCC (MS 96 months with surgery vs. 108 months without, *P* = 0.796). Figure 1B demonstrates the effect of RT on AC (MS 110 months with RT vs. 76 months without, *P* < 0.0001), but revealed a relatively greater magnitude of improvement in OS in the SCC group (MS 135 months with RT vs. 51 months without, *P* < 0.0001).

Other notable prognostic factors were associated with survival, as quantitated in Table 2. Stage of presentation had a statistically significant impact on OS in both groups (*P* < 0.0001). Though there were statistically more high‐grade tumors in the SCC group, grade influenced OS in AC (*P* < 0.0001), but not SCC (*P* = 0.900). Younger age and female gender were associated with improved outcomes as well; age in both SCC/AC (*P* < 0.0001), and gender in SCC (*P* < 0.0001) but not AC (*P* = 0.332). Lastly, tumor size was a relevant factor in both groups (*P* < 0.0001), especially SCC.

**Table 2:** Median overall survivals of rectal adenocarcinomas versus squamous cell carcinomas, stratified for several variables, corresponding to Figure 1 and Figures S1–S5

	Squamous cell carcinoma		Adenocarcinoma	
	Median OS (mo)(95% CI)	Log‐rank *P* value	Median OS (mo)(95% CI)	Log‐rank *P* value
Surgery		0.796		<0.0001
Yes	96 (69–123)		106 (104–109)	
No	108 (83–139)		22 (21–24)	
RT		<0.0001		<0.0001
Yes	135 (102–147)		110 (106–113)	
No	51 (22–70)		76 (74–79)	
Stage		<0.0001		<0.0001
Local	119 (105–139)		115 (112–119)	
Regional	40 (30–78)		76 (74–79)	
Grade		0.900		<0.0001
Low	107 (93–136)		99 (97–102)	
High	95 (75–136)		68 (64–73)	
Age		<0.0001		<0.0001
≤65	136 (119–167)		Not reached	
>65	39 (29–59)		58 (57–60)	
Gender		<0.0001		0.332
Male	123 (105–149)		98 (94–101)	
Female	57 (36–93)		93 (90–95)	
Tumor size		<0.0001		<0.0001
≤3.9 cm	115 (111–119)		129 (126–132)	
>3.9 cm	85 (81–88)		110 (102–118)	

OS, overall survival; RT, radiotherapy; CI, confidence intervals.

Using the aforementioned prognostic factors, low‐, intermediate‐, and high‐risk groups were then constructed for each histologic type. SCC risk factors included stage (regional disease), gender (male), and age (>65 years), whereas those for AC included stage (regional disease), grade (high), and age (>65 years). The high‐risk group for each histologic subtype required all three risk factors, the intermediate‐risk group 1–2 risk factors, and the low‐risk required the absence of all three.

**Figure 2:**
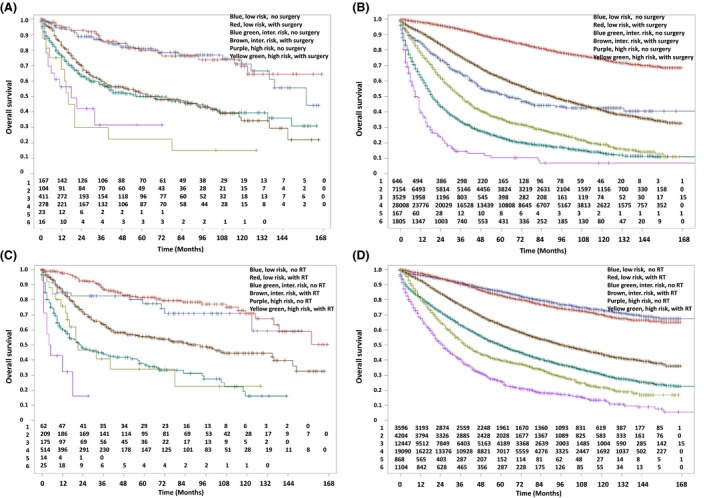
(A). Comparison of overall survival in rectal squamous cell carcinoma patients, as stratified for risk status and receipt of surgery.(B). Comparison of overall survival in rectal adenocarcinoma patients, as stratified for risk status and receipt of surgery. (c).. Comparison of overall survival in rectal squamous cell carcinoma patients, as stratified for risk status and receipt of RT. RT, radiotherapy.(D).. Comparison of overall survival in rectal adenocarcinoma patients, as stratified for risk status and receipt of RT. RT, radiotherapy.

Figure 2 and Table 3 compare SCC and AC in their respective risk groups. In SCC, surgery did not seem to be associated with higher OS among each risk group (*P* > 0.05 for all groups), whereas there was a strong association in AC cases (*P* < 0.0001 for all groups). When assessing the effect of RT, however, both SCC and AC displayed higher OS when RT was performed in both intermediate‐ (*P* < 0.0001 for both SCC and AC) and high‐risk (*P* = 0.007 for SCC, *P* < 0.0001 for AC) subgroups. An alternative presentation of the data based on risk groups in all patients, those that underwent surgery, and those that did not undergo surgery are presented in Figures S1–S3, respectively, corresponding to Table S1.

**Table 3:** Median overall survivals of rectal adenocarcinomas versus squamous cell carcinomas, stratified for treatment intervention and risk groups, corresponding to Figure 2

	Low Risk		Intermediate Risk		High Risk	
	MS (mo)(95% CI)	Log‐rank*P* value	MS (mo)(95% CI)	Log‐rank *P* value	MS (mo)(95% CI)	Log‐rank *P* value
SCC						
Surgery	Not reached	0.853	64 (42–95)	0.459	16 (5–40)	0.824
No surgery	158 (136–165)		71 (41–97)		18 (4–72)	
RT	Not reached	0.219	88 (64–135)	<0.0001	21 (14–78)	0.007
No RT	Not reached		23 (15–43)		4.5 (1–18)	
AC						
Surgery	Not reached	<0.0001	90 (88–93)	<0.0001	37 (33–39)	<0.0001
No surgery	64 (50–78)		19 (18–21)		9 (7–11)	
RT	Not reached	0.036	97 (94–100)	<0.0001	39 (36–43)	<0.0001
No RT	Not reached		60 (58–62)		25 (22–29)	

MS, median survival; CI, confidence interval; SCC, squamous cell carcinoma; AC, adenocarcinoma; RT, radiotherapy.

### Prognostic factors

Prognostic factors for 5‐year OS included histology (AC vs. SCC), surgery (with vs. without), RT (with vs. without), stage (regional vs. local), age (>65 vs. ≤65), gender (male vs. female), and race (white vs. non‐white). Favorable prognostic factors for all‐comers included SCC histology, having undergone surgery, receipt of RT, local stage, younger age, female gender, and white race.

After adjusting for relevant prognostic factors in all patients, patients with AC were less likely to survive ≥5 years, relative to their SCC counterparts (Fig. S4; odds ratio [OR]: 0.838; 95% CI: 0.720–0.974). This was also true in patients who had not undergone surgery (Fig. S5; OR: 0.533; 95% CI: 0.422–0.674). However, for patients who had undergone surgery, patients with AC were more likely to survive ≥5 years, relative to their SCC counterparts (Fig. S6; OR: 1.516; 95% CI: 1.227–1.873).

### Propensity score matching analysis

Propensity score matching was used to select AC and SCC cases with equal distributions of all observed covariates, in effort to examine the associations of survival with histology alone. Table S2 shows that patient, tumor, and treatment characteristics of the 907 patients in each group of the matched population were statistically insignificant in all analyzed variables except year interval of diagnosis. In the matched data, AC was significantly associated with increased risk of overall death as compared to SCC in both univariate (HR = 1.156; 95% CI: 1.009–1.323) and multivariate (HR = 1.228; 95% CI: 1.021–1.475) Cox proportional hazard regression models (Table 4).

**Table 4:** Cox proportional hazards models built on propensity score‐matched data

Variable	Univariate model		Multivariate modelSelection=stepwise	
	HR (95% CI)	*P* value Chi‐square	HR (95% CI)	*P* value Chi‐square
Histology				
SCC	Reference		Reference	
AC	1.156 (1.009–1.323)	0.0363	1.270 (1.057–1.527)	0.011
Age				
19–65	Reference		Reference	
66–100	2.243 (1.959–2.568)	<0.0001	2.402 (1.998–2.887)	<0.0001
Gender				
Male	1.256 (1.093–1.443)	0.0013	1.363 (1.132–1.640)	0.001
Female	Reference		Reference	
Tumor size				
>3.9 cm	1.739 (1.445–2.094)	<0.0001	Not included due to missing data (40%)	
≤3.9 cm	Reference			
Race				
White	0.994 (0.827–1.194)	0.949		
Non‐white	Reference			
Stage				
Localized	Reference		Reference	
Regional	1.460 (1.272–1.676)	<0.0001	1.544 (1.342–1.777)	<0.0001
Grade				
1 and 2	Reference			
3 and 4	1.277 (1.114–1.464)	0.0001		
Surgery				
Yes	0.609 (0.530–0.700)	<0.0001	0.603 (0.498–0.731)	<0.0001
No	Reference		Reference	
Radiation therapy				
Yes	0.653 (0.565–0.755)	<0.0001	0.533 (0.462–0.616)	<0.0001
No	Reference		Reference	
Year of diagnosis				
1998–2003	Reference			
2004–2011	1.122 (0.972–1.296)	0.065		

Only statistically significant variables are shown in the table. HR, hazard ratio; CI, confidence interval; SCC, squamous cell carcinoma; AC, adenocarcinoma.

## Discussion

Diagnoses of rectal SCC are rising over time, despite high‐volume evidence regarding treatment options and modalities. Despite the rarity of rectal SCC, this is the first known study to study a large population‐based sample of patients (*n* = 999) with rectal SCC. To the best of our knowledge, the second‐largest report evaluated just 107 patients, with details unavailable owing to the non‐English language nature of the publication 18. We found that the majority of patients with SCC were treated with RT, whereas the majority of patients with AC were treated with surgery; the patients with SCC showed a significantly superior OS. In addition to SCC histology, other factors associated with higher survival in the entire cohort (AC + SCC) included receipt of RT, undergoing surgery, local stage, younger age, female gender, and white race. When stratified by histology, receipt of surgery and race were no longer prognostic factors for survival in patients with SCC histology. Propensity score matching also illustrated that histology (SCC greater than AC) was a significant associate of survival after controlling for nearly all remaining variables.

Although treatment for AC most commonly involves surgery alone for early localized disease and chemoradiation therapy followed by surgical resection (if possible) followed by adjuvant chemotherapy for T3/T4, node‐positive, and technically/medically unresectable disease 19, guidelines for rectal SCC are less clear owing to a lack of high‐volume data. Surgical resection is a consideration 20, but increasing numbers of recent studies are also finding merit to treat with definitive CRT using the anal SCC paradigm 21, 22. Along with the data herein, several series have shown high clinical response as well as clinical complete response rates from 63% to 100% along with 5‐year overall survival, disease‐free survival, and disease‐specific survival rates of 81%, 72%, and 88%, respectively 9, 23, 24, 25, 26, 27, 28.

There are several limitations of our analysis. In addition to the inherently retrospective nature of SEER studies as well as individualized follow‐up, it must be prominently mentioned that causation can neither be stated nor implied with these data, especially regarding treatment interventions and survival. Additionally, lack of chemotherapy records makes these data somewhat incomplete. It would be helpful to know the response of rectal SCCs to chemotherapy in efforts to escalate or deescalate therapy accordingly. Additionally, there could have been a degree of selection bias in those patients that did not receive surgery, potentially owing to additional comorbidities impacting outcomes. A noted selection bias was the inclusion of nonmetastatic cases, but as previously noted, most metastatic cases receive RT only for palliation; these patients were hence beyond the scope of our specific clinical question. Removal of cases with missing data could also introduce bias, but this proportion was low; moreover, it was a necessity to have basic data for subgroup/prognostic analyses. Furthermore, it cannot be understated that miscoding is a limitation of any population‐based study, even regardless of the specific database used. It can never be known whether every patient in this study had true rectal SCC, because many rectal SCC cases are the result of misdiagnosis of anal squamous cell carcinomas with proximal extension into the rectum 8. Whether there are true prognostic differences between “true” SCCs of the rectum and anus remains to be addressed, but other groups have been able to find outcome differences between anal SCCs and anal ACs 29.

There remains much need for further elucidation. It would be important to know the clinical and pathologic response rates for rectal SCC with CRT or RT alone, as well as patterns of failure, which can only be possible using more detailed clinical data than what is available in SEER. Furthermore, it remains to be seen whether subsets of rectal SCC (low‐risk subgroup) with the best prognosis could be afforded deescalated therapy (and vice‐versa with high‐risk groups), with appropriate surgical salvage in those specific circumstances. Lastly, a study has also found the presence of the SCC antigen, which was present in 50% of studied patients, but decreased with treatment and as correlated with complete response 23. The value of such a marker is unknown, is far from validated, and also must be further investigated.

## Conclusions

In the largest analysis of rectal SCC to date, nonsurgical, RT‐based treatment is recommended. Recognizing that prospective data for this rare tumor will be difficult to accrue, future population‐based analyses should continue to build on the implications of results in this SEER analysis.

## Conflicts of Interest

The authors all declare that conflicts of interest do not exist.

## Supporting information


**Figure S1**. Comparison of overall survival between all patients with rectal AC and SCC using risk stratification. SCC, squamous cell carcinoma; AC, adenocarcinoma.

**Figure S2**. Comparison of overall survival between patients with rectal AC and SCC who underwent surgery, using risk stratification. SCC, squamous cell carcinoma; AC, adenocarcinoma.

**Figure S3.** Comparison of overall survival between patients with rectal AC and SCC who did not undergo surgery, using risk stratification. SCC, squamous cell carcinoma; AC, adenocarcinoma.

**Figure S4**. Analysis of factors influencing overall survival rates in all patients. Numerical odds ratios for each variable are as follows: histology (<1, favor SCC; >1, favor AC), surgery (<1, favor no surgery; >1, favor surgery), RT (<1, favor no RT; >1, favor RT), stage (<1, favor local; >1, favor regional), age (<1, favor ≤ 65 years; >1, favor > 65 years), gender (<1, favor female; >1, favor male), race (<1, favor non‐white; >1, favor white), grade (<, favor low‐grade; >1, favor high‐grade). SCC, squamous cell carcinoma; AC, adenocarcinoma; RT, radiotherapy; Reg, regional; Loc, local; M, male; F, female; W, while; NW, non‐white; H, high‐grade; L, low‐grade.

**Figure S5**. Analysis of factors influencing overall survival rates in patients without surgery. Numerical odds ratios for each variable are as follows: histology (<1, favor SCC; >1, favor AC), RT (<1, favor no RT; >1, favor RT), stage (<1, favor local; >1, favor regional), age (<1, favor ≤ 65 years; >1, favor > 65 years), gender (<1, favor female; >1, favor male), race (<1, favor non‐white; >1, favor white), grade (<, favor low‐grade; >1, favor high‐grade). SCC, squamous cell carcinoma; AC, adenocarcinoma; RT, radiotherapy; Reg, regional; Loc, local; M, male; F, female; W, while; NW, non‐white; H, high‐grade; L, low‐grade.

**Figure S6**. Analysis of factors influencing overall survival rates in patients with surgery. Numerical odds ratios for each variable are as follows: histology (<1, favor SCC; >1, favor AC), RT (<1, favor no RT; >1, favor RT), stage (<1, favor local; >1, favor regional), age (<1, favor ≤ 65 years; >1, favor > 65 years), gender (<1, favor female; >1, favor male), race (<1, favor non‐white; >1, favor white), grade (<, favor low‐grade; >1, favor high‐grade). SCC, squamous cell carcinoma; AC, adenocarcinoma; RT, radiotherapy; Reg, regional; Loc, local; M, male; F, female; W, while; NW, non‐white; H, high‐grade; L, low‐grade.

**Table S1**. Median overall survivals of rectal adenocarcinomas versus squamous cell carcinomas stratified for treatment intervention and risk groups, corresponding to Figures S6–S8. MS, median survival; CI, confidence interval; SCC, squamous cell carcinoma; AC, adenocarcinoma; RT, radiotherapy.
**Table S2.** Propensity score analysis demonstrating characteristics of the 1:1 matched population. SCC, squamous cell carcinoma; AC, adenocarcinoma.
