# Supplementary figures and images for: Comparison of outcomes between rectal squamous cell carcinoma and adenocarcinoma
Source: Cancer Med. 2016 Oct 26;5(12):3394–402. doi: 10.1002/cam4.927 (PMC5224838; doi:10.1002/cam4.927)

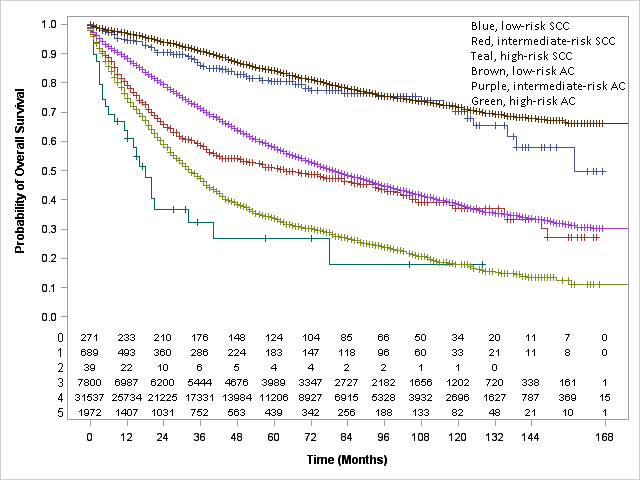

Supplement: Supplementary file 1 — Figure S1. Comparison of overall survival between all patients with rectal AC and SCC using risk stratification. SCC, squamous cell carcinoma; AC, adenocarcinoma. [file CAM4-5-3394-s001.tiff]

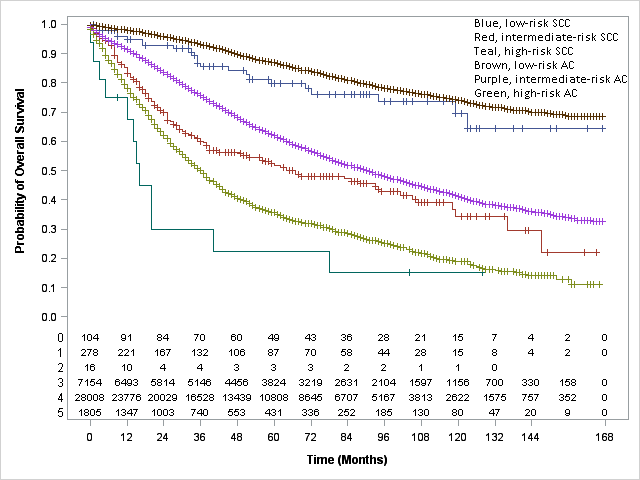

Supplement: Supplementary file 2 — Figure S2. Comparison of overall survival between patients with rectal AC and SCC who underwent surgery, using risk stratification. SCC, squamous cell carcinoma; AC, adenocarcinoma. [file CAM4-5-3394-s002.tiff]

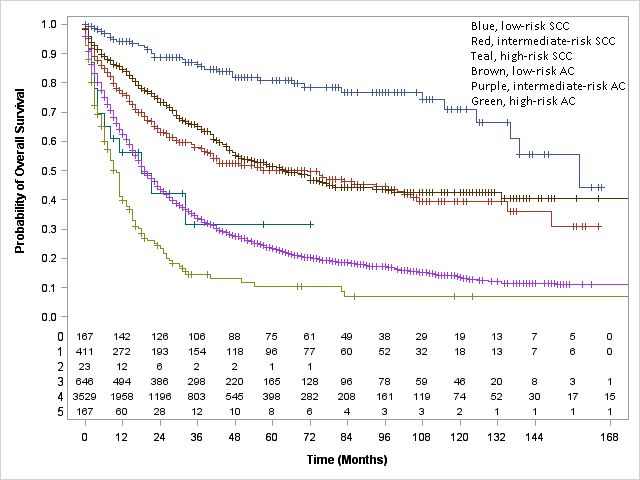

Supplement: Supplementary file 3 — Figure S3. Comparison of overall survival between patients with rectal AC and SCC who did not undergo surgery, using risk stratification. SCC, squamous cell carcinoma; AC, adenocarcinoma. [file CAM4-5-3394-s003.tiff]

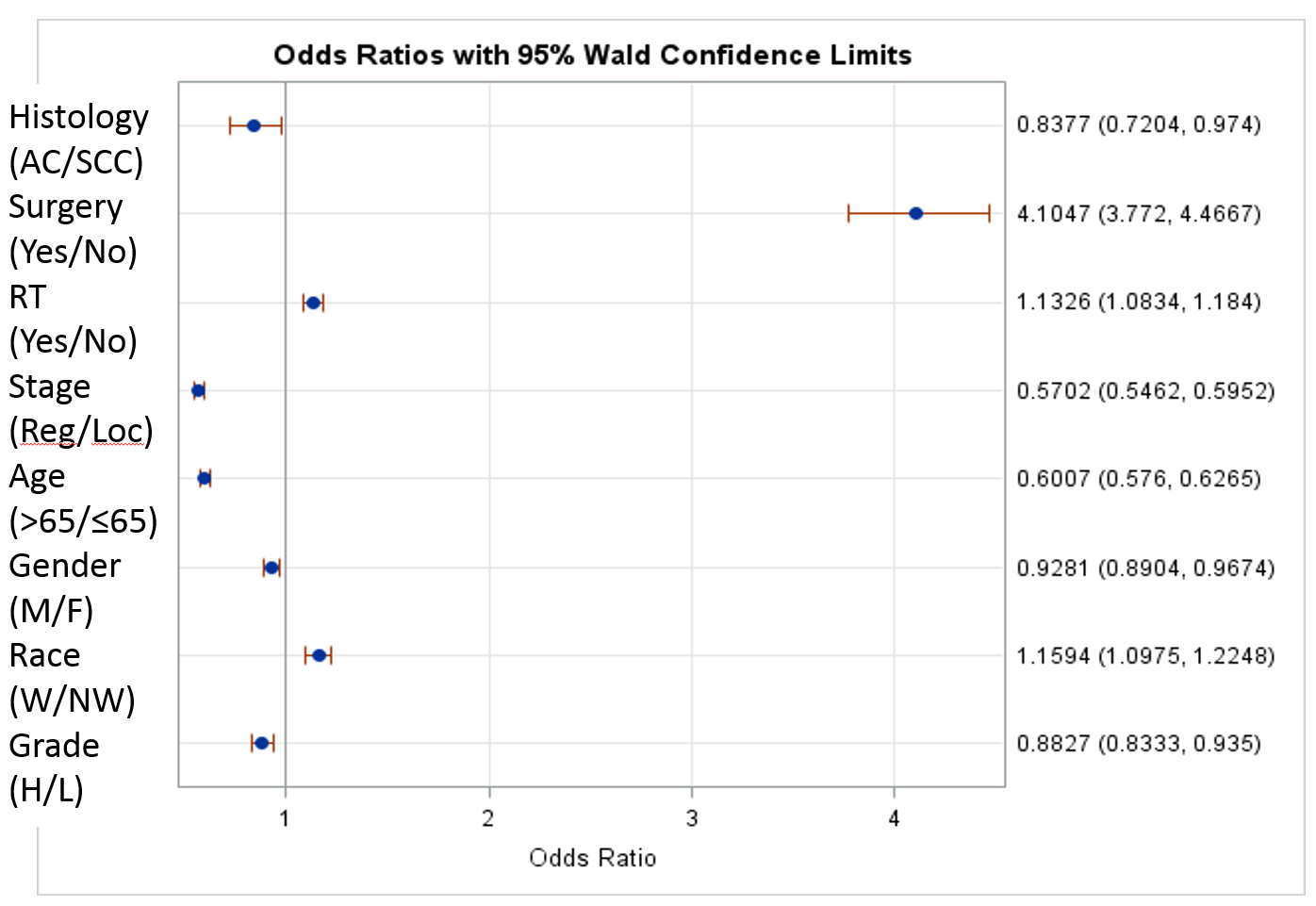

Supplement: Supplementary file 4 — Figure S4. Analysis of factors influencing overall survival rates in all patients. Numerical odds ratios for each variable are as follows: histology (<1, favor SCC; >1, favor AC), surgery (<1, favor no surgery; >1, favor surgery), RT (<1, favor no RT; >1, favor RT), stage (<1, favor local; >1, favor regional), age (<1, favor ≤ 65 years; >1, favor > 65 years), gender (<1, favor female; >1, favor male), race (<1, favor non‐white; >1, favor white), grade (<, favor low‐grade; >1, favor high‐grade). SCC, squamous cell carcinoma; AC, adenocarcinoma; RT, radiotherapy; Reg, regional; Loc, local; M, male; F, female; W, while; NW, non‐white; H, high‐grade; L, low‐grade. [file CAM4-5-3394-s004.tiff]

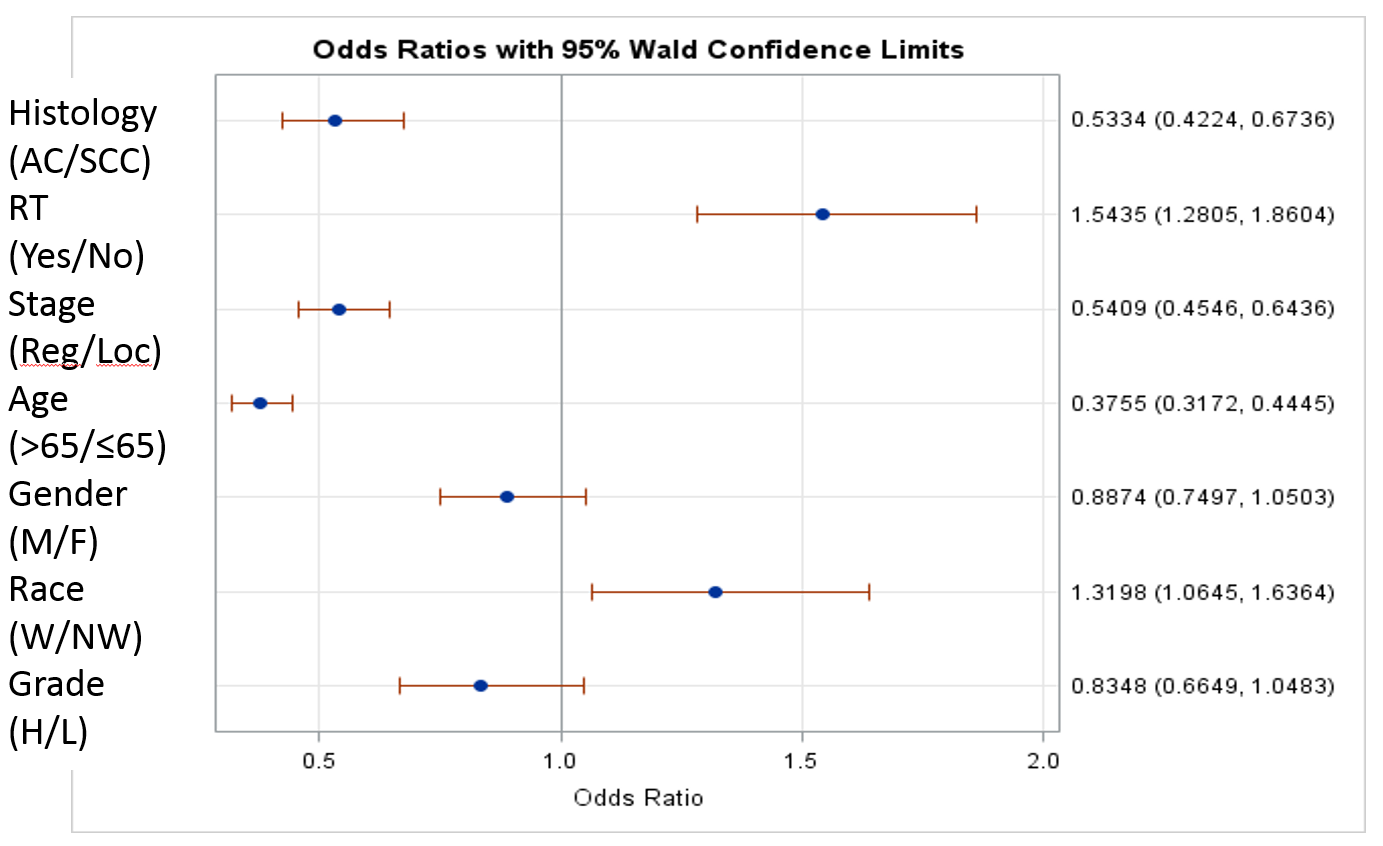

Supplement: Supplementary file 5 — Figure S5. Analysis of factors influencing overall survival rates in patients without surgery. Numerical odds ratios for each variable are as follows: histology (<1, favor SCC; >1, favor AC), RT (<1, favor no RT; >1, favor RT), stage (<1, favor local; >1, favor regional), age (<1, favor ≤ 65 years; >1, favor > 65 years), gender (<1, favor female; >1, favor male), race (<1, favor non‐white; >1, favor white), grade (<, favor low‐grade; >1, favor high‐grade). SCC, squamous cell carcinoma; AC, adenocarcinoma; RT, radiotherapy; Reg, regional; Loc, local; M, male; F, female; W, while; NW, non‐white; H, high‐grade; L, low‐grade. [file CAM4-5-3394-s005.tiff]

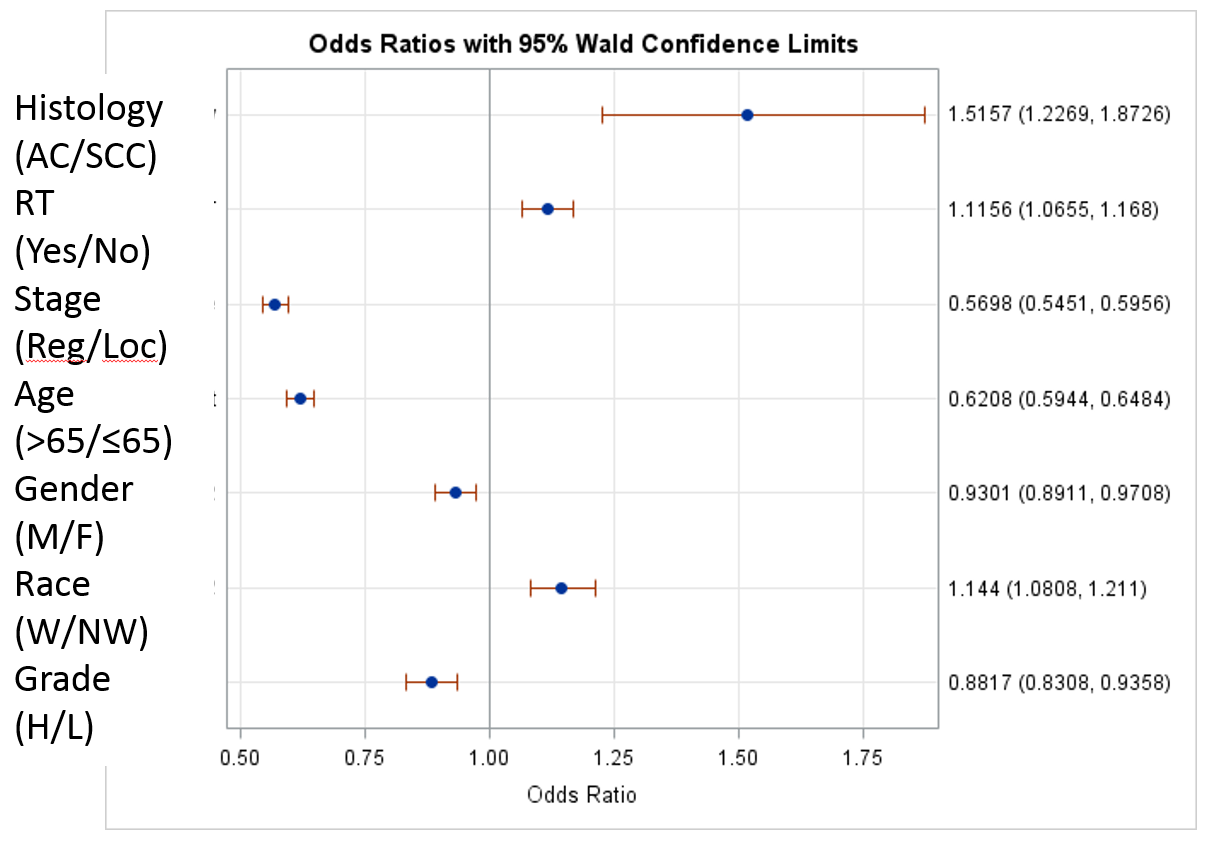

Supplement: Supplementary file 6 — Figure S6. Analysis of factors influencing overall survival rates in patients with surgery. Numerical odds ratios for each variable are as follows: histology (<1, favor SCC; >1, favor AC), RT (<1, favor no RT; >1, favor RT), stage (<1, favor local; >1, favor regional), age (<1, favor ≤ 65 years; >1, favor > 65 years), gender (<1, favor female; >1, favor male), race (<1, favor non‐white; >1, favor white), grade (<, favor low‐grade; >1, favor high‐grade). SCC, squamous cell carcinoma; AC, adenocarcinoma; RT, radiotherapy; Reg, regional; Loc, local; M, male; F, female; W, while; NW, non‐white; H, high‐grade; L, low‐grade. [file CAM4-5-3394-s006.tiff]
