# Supplementary material for: Comparison of outcomes between rectal squamous cell carcinoma and adenocarcinoma
Source: Cancer Med. 2016 Oct 26;5(12):3394–402. doi: 10.1002/cam4.927 (PMC5224838; doi:10.1002/cam4.927)
Supplement: Supplementary file 7 — Table S1. Median overall survivals of rectal adenocarcinomas versus squamous cell carcinomas stratified for treatment intervention and risk groups, corresponding to Figures S6–S8. MS, median survival; CI, confidence interval; SCC, squamous cell carcinoma; AC, adenocarcinoma; RT, radiotherapy. Table S2. Propensity score analysis demonstrating characteristics of the 1:1 matched population. SCC, squamous cell carcinoma; AC, adenocarcinoma. [file CAM4-5-3394-s007.docx]

**Supplementary Table 1**. Median overall survivals of rectal adenocarcinomas versus squamous cell carcinomas, stratified for treatment intervention and risk groups, corresponding to Supplementary Figures 6-8. MS, median survival; CI, confidence interval; SCC, squamous cell carcinoma;

AC, adenocarcinoma; RT, radiotherapy.

|  | | **Low Risk** | | **Intermediate Risk** | | **High Risk** | |
| --- | --- | --- | --- | --- | --- | --- | --- |
|  |  | **MS (mo)**  **(95% CI)** | **Log-rank**  **P value** | **MS (mo)**  **(95% CI)** | **Log-rank P value** | **MS (mo)**  **(95% CI)** | **Log-rank P value** |
| **All** | SCC | 158 (136-167) | 0.146 | 64 (43-88) | <0.0001 | 18 (8-32) | 0.046 |
|  | AC | Not reached |  | 80 (78-81) |  | 33 (31-36) |  |
| **Surgery** | SCC | Not reached | 0.140 | 64 (42-95) | 0.0002 | 16 (5-40) | 0.12 |
|  | AC | Not reached |  | 90 (88-93) |  | 37 (33-39) |  |
| **No Surgery** | SCC | 158 (136-165) | <0.0001 | 71 (41-97) | <0.0001 | 18 (4-72) | 0.05 |
|  | AC | 64 (50-78) |  | 19 (18-21) |  | 9 (7-11) |  |

**Supplemental Table 2.** Propensity score analysis demonstrating characteristics of the 1:1 matched population. SCC, squamous cell carcinoma; AC, adenocarcinoma.

|  |  | Adenocarcinoma  N (%) | Squamous Cell Carcinoma  N (%) | Chi^2^ P-value |
| --- | --- | --- | --- | --- |
|  | **Number of Patients** | 907 | 907 |  |
| **Age** |  |  |  |  |
|  | Median (range) | 61 (20-99) | 62 (23-96) |  |
|  | 19-65 | 556 (61) | 529 (58) | 0.196 |
|  | 66-100 | 351 (39) | 378 (42) |  |
| **Gender** |  |  |  | 0.960 |
|  | Male | 307 (34) | 308 (34) |  |
|  | Female | 600 (66) | 599 (66) |  |
| **Race** |  |  |  | 0.523 |
|  | White | 766 (84) | 756 (83) |  |
|  | Non-White | 141 (16) | 151 (17) |  |
| **Tumor Size** |  |  |  | 0.148 |
|  | > 3.9 cm | 288 (32) | 327 (36) |  |
|  | ≤ 3.9 cm | 251 (28) | 240 (26.5) |  |
|  | Missing | 368 (40) | 340 (37.5) |  |
| **Stage** |  |  |  | 0.772 |
|  | Localized | 557 (61) | 563 (62) |  |
|  | Regional | 350 (39) | 344 (38) |  |
| **Grade** |  |  |  | 0.775 |
|  | 1 and 2 | 539 (59) | 533 (59) |  |
|  | 3 and 4 | 368 (41) | 374 (41) |  |
| **Surgery** |  |  |  | 0.813 |
|  | Yes | 398 (44) | 393 (43) |  |
|  | No | 509 (56) | 514 (57) |  |
| **Radiation Therapy** |  |  |  | 0.710 |
|  | Yes | 668 (74) | 661 (73) |  |
|  | No | 239 (26) | 246 (27) |  |
| **Year of diagnosis** |  |  |  | <0.0001 |
|  | 1998-2003 | 306 (34) | 401 (44) |  |
|  | 2004-2011 | 601 (66) | 506 (56) |  |
